# Paracrystals of STAT proteins and their dissolution by SUMO: How reduced transcription factor solubility increases cytokine signaling

**DOI:** 10.18632/oncotarget.307

**Published:** 2011-07-26

**Authors:** Mathias Droescher, Andreas Begitt, Uwe Vinkemeier

**Affiliations:** ^1^School of Biomedical Sciences, Nottingham University Medical School, Nottingham NG7 2UH, United Kingdom

The cell nucleus is a highly organized organelle. In the past decade, a multitude of nuclear substructures and their possible role in gene regulation were identified [1]. We are studying STAT transcription factors, which comprise a structurally and functionally conserved family of proteins with indispensable roles in cytokine signaling. Cytokine binding to specific membrane receptors first activates receptor-associated JAK tyrosine kinases, which activate STAT proteins by phosphorylating a single C-terminal tyrosine. The activated STATs possess DNA-binding activity and evoke transcriptional responses to cytokines [2]. We discovered that activated STAT dimers can polymerize and assemble paracrystalline arrays in the nucleus of cytokine-stimulated cells [3]. Hepatocytes are an example, which harbor STAT3 paracrystals during systemic bacterial infection. Paracrystal formation is governed by the principle of self-organization [4] and necessitates a threshold concentration of the activated STAT [3]. STAT paracrystals are dynamic and reversible structures that form randomly and independently of specific nuclear components. Although paracrystal formation decreased the abundance of the soluble and hence transcriptionally active STAT, its transcriptional response was enhanced [3].

Paracrystal formation seems to be an inherent property of the STAT family, but STAT1 normally does not form these structures. We could show that this is due to the unique ability of STAT1 among the STATs to conjugate to small ubiquitin-like modifier (SUMO) [3]. SUMO conjugation can lead to the association of proteins, e.g. PML bodies. In contrast, sumoylation dissociated STAT1 paracrystals. SUMO facilitated the generation of a competitive polymerization inhibitor, namely semi-phosphorylated STAT1 dimers. These in turn competed with their fully phosphorylated counterparts and interfered with their polymerization into paracrystals. Importantly, the competition itself and hence the prevention of paracrystals was achieved entirely independent of SUMO's continued presence [3].

STAT paracrystal dissolution by SUMO thus constitutes a showcase example of how a disproportionately small SUMO-modified fraction determines the behavior of the entire pool of molecules, also known as the “SUMO enigma” [5]. We propose that in many cases the solution to it entails the mutual exclusion of SUMO conjugation and another protein modification -tyrosine phosphorylation in the case of STAT1 [3]. Thus, SUMO acts as a bulky obstacle to another modifying event, e.g. ubiquitination or proteolytic cleavage. In this way SUMO conjugation facilitates the generation of molecules that have different properties than those that have not been SUMO-modified. Importantly, these molecules can subsequently act as competitive inhibitors of protein interactions entirely independent of SUMO. We propose that these mechanisms are underlying several SUMO-dependent protein regulatory events, one of which is control of protein polymerization and hence solubility.

We then explored the physiological consequences associated with paracrystal dissolution by a small SUMO-modified STAT1 fraction. By using knock-in mice expressing exclusively SUMO-free STAT1 we could show that paracrystals demonstrated a buffer property for activated STAT1, such that SUMO-mediated paracrystal dispersal profoundly accelerated the dephosphorylation of STAT1 [6]. Accordingly, the curtailed STAT1 activity in the nucleus resulted in diminished transcription of IFNγ-responsive genes. Furthermore, while the already known negative regulators of STAT1 function in a feedback manner, sumoylation acts as a permanent attenuator of IFNγ signaling. This in turn protected cells from hyperresponsiveness towards this cytokine and its potentially self-destructive consequences [6].

In summary, we present STAT paracrystals as a model for the regulation of protein solubility by SUMO. We propose that the mechanisms underlying this phenomenon are of importance for understanding how SUMO generally regulates protein function. Paracrystals were identified as reservoirs for activated STATs which play a central role in the positive regulation of STAT-signaling. The SUMO conjugation of STAT1 may interfere with the polymerization of other STAT family members too, providing a structural basis for the cross-regulation of multiple cytokine signaling pathways by IFNγ. Furthermore, in conjunction with its great significance for interferon signaling, the modulation of STAT1 SUMO conjugation thus becomes feasible for pharmacological interventions with therapeutic potential in inflammatory and immune disorders.
